# Dataset for supporting a modular autoinduction device for control of gene expression in *Bacillus subtilis*

**DOI:** 10.1016/j.dib.2020.105736

**Published:** 2020-05-21

**Authors:** Graciely Gomes Correa, Milca Rachel da Costa Ribeiro Lins, Bruna Fernandes Silva, Gabriela Barbosa de Paiva, Vitoria Fernanda Bertolazzi Zocca, Nathan Vinicius Ribeiro, Flavio Pereira Picheli, Matthias Mack, Danielle Biscaro Pedrolli

**Affiliations:** a Universidade Estadual Paulista (UNESP), School of Pharmaceutical Sciences, Department of Bioprocess Engineering and Biotechnology, Rodovia Araraquara-Jau km1, 14800-903 Araraquara, Brazil; b Mannheim University of Applied Sciences, Institute for Technical Microbiology, Paul-Wittsack-Str. 10, 68163, Mannheim, Germany

**Keywords:** Autoinduction, Quorum sensing, LuxR/I, Dynamic regulation, *Bacillus subtilis*, Synthetic biology

## Abstract

Modular and tuneable genetic tools for Metabolic Engineering fuels the development of chassis for the efficient production of biocompounds at industrial scale. We have constructed an autoinduction device for gene expression in *Bacillus subtilis* based on the LuxR/I quorum sensing system [1]. Here, we present raw and processed data regarding to *B. subtilis* growth measured as OD_600_, performed in three different scales: microcultivation on 96-well plates (200 µL), test tubes (12 mL), and Erlenmeyer flasks (50 mL). We also present raw and processed data on gene expression measured as GFP fluorescence (485/535 nm), luminescence and riboflavin production. Measurements were performed on a microplate reader Tecan 200 PRO (iControl software) and on spectrophotometer (Thermo Fisher Scientific GENESYS 10S UV-Vis). Processed data are presented as product/OD_600_, maximum and minimum promoter activity, fold of induction, and the induction OD_600_.

Specifications table

**Table:** 

Subject	Biochemistry, Genetics and Molecular Biology (General)
**Specific subject area**	Synthetic Biology. Development of autoinduction devices as a toolbox for Metabolic Engineering
**Type of data**	Datasheets (excel format)
**How data were acquired**	The Microplate reader Tecan Infinite 200 Pro (Tecan, Männedorf, Switzerland) was used to measure optical density, luminescence and fluorescence. The equipment runs the i-Control (1.11.1.0 version) software and Microsoft Excel 2010. A Spectrophotometer Thermo Fisher Scientific GENESYS 10S UV-Vis was used to measure optical density in the scale-up process. Data were processed using the Microsoft Excel 2010 and Spyder (Python 3.7) programs.
**Data format**	Raw Processed
**Parameters for data collection**	Bacterial cultures were carried out at 37°C in all scales. 96-well microplates were maintained at constant orbital agitation at 143 rpm (amplitude 6 mm), while test tubes and flasks were shaken at 220 rpm (orbit 1.9 cm). Culture optical density was measured at 600 nm. Luminescence emission was acquired using an integration time of 1,000 ms. For GFP fluorescence measurements, excitation was set to 485 nm and emission was detected at 535 nm, using a gain 100. Riboflavin was also quantified by fluorescence measurement using an excitation at 445 nm and emission at 524 nm, with a gain 80.
**Description of data collection**	Data were collected directly during growth of the microorganism on a 96-well microplate reader. For cultivations carried out in test tubes or flasks, samples were withdrawn periodically and measured in the microplate reader or in the spectrophotometer, as indicated.
**Data source location**	Universidade Estadual Paulista (UNESP), School of Pharmaceutical Sciences, Department of Bioprocess Engineering and Biotechnology, Rodovia Araraquara-Jau km1, 14800-903 Araraquara, Brazil Latitude and longitude for collected samples/data: 21.8024° S, 48.1916° W
**Data accessibility**	Repository name: Mendeley Data Data identification number: 10.17632/79mm425n5v.2 Direct URL to data: http://dx.doi.org/10.17632/79mm425n5v.2
**Related research article**	G.G. Correa, M.R.C.R. Lins, B.F. Silva, G.B. Paiva, V.F.B. Zocca, N.V. Ribeiro, F.P. Picheli, M. Mack, D.B. Pedrolli, A modular autoinduction device for control of gene expression in *Bacillus subtilis,* Metab. Eng., 2020. https://doi.org/10.1016/j.ymben.2020.03.012

## Value of the Data

• These data show how our autoinduction devices perform over single copy target genes in the bacterial chromosome.
• These data support further progress in engineering *B. subtilis* as an efficient chassis for industrial bioprocesses.
• These data will be useful for other researchers willing to use and/or further develop the autoinduction devices designed for *B. subtilis*.
• These data could help other researchers to develop similar devices for other bacterial species.

## 1 Data Description

This article presents the dataset for supporting analysis of gene expression in *B. subtilis* as well as ways to assess the optimization of bacterial promoters. We present a modular autoinduction device for control of gene expression at single copy in the genome. We make data available on simultaneous measurement of growth, and bioluminescence or fluorescence directly in a microplate reader. These data are part of the supplementary information for the paper “A modular autoinduction device for control of gene expression in *Bacillus subtilis”* [1].

The Supplementary Data 1 presents culture optical density (OD_600_) and luminescence raw data measured during growth in a 96-well microplate. The first worksheet handles OD_600_ data: raw data (biological triplicate), processed data discounting blank, mean and standard deviation. The second worksheet handles Luminescence data: raw data (biological triplicate), normalized luminescence to OD_600_ discounting blank, replace values below 0 by 0, mean and standard deviation. The third worksheet shows processed data for fold change, minimum and maximum promoter activities, and standard deviations. The fourth worksheet shows the AHL (N-(3-Oxohexanoyl)-L-homoserine lactone)-dependent induction of gene expression test, in which AHL was added to the culture medium in different concentrations, the engineered strains luxR-R4 is used and the data present: raw data (biological triplicate) for OD_600_ and luminescence, normalized luminescence to OD_600_, mean and standard deviation.

The Supplementary Data 2 presents growth data and luminescence evaluation in the test tubes. The first worksheet handles OD_600_ raw data (biological triplicate), processed data discounting blank, mean and standard deviation, and conversion of microplate reader (200 µL) measurement to spectrophotometer measurement (10 mm pathlength). The second worksheet handles luminescence raw data (biological triplicate), luminescence discounting blank, replace values below 0 by 0, mean and standard deviation. The third worksheet shows normalized luminescence to OD_600_, mean and standard deviation, processed data for fold change, minimum and maximum promoter activities. The fourth worksheet shows processed data for fold change.

The Supplementary Data 3 presents data on growth and GFP fluorescence for cultivations carried out in three different culture media (high sugar complex medium – PW, Luria Bertani – LB, and Mineral medium - MM), in 96-well microplate. The first, second and third worksheets handle the data in PW medium. The first, OD_600_ raw data (biological triplicate), processed data discounting blank, mean and standard deviation. The second worksheet handles Fluorescence raw data (biological triplicate), normalized fluorescence to OD_600_ discounting blank, replace values below 0 by 0, mean and standard deviation. The third worksheet shows processed data for fold change, fold change mean, and standard deviation. The fourth worksheet shows OD_600_ in MM and LB media: raw data (biological triplicate), processed data discounting blank, mean and standard deviation. The fifth, handles fluorescence in MM and LB media: raw data (biological triplicate), normalized fluorescence to OD_600_ discounting blank, mean and standard deviation, processed data for fold change, minimum and maximum promoter activities.

The Supplementary Data 4 presents data on growth and Riboflavin production in a 96-well microplate and in Erlenmeyer flasks. The first worksheet handles OD_600_ and Riboflavin in microplate reader, OD_600_ raw data (biological triplicate), mean and standard deviation, fluorescence, mean and standard deviation, normalized fluorescence to OD_600,_ mean and standard deviation. The second worksheet handles Growth data and Riboflavin evaluation in Erlenmeyer: OD_600_ raw data, mean and standard deviation, fluorescence, normalized riboflavin concentration to OD_600,_ mean and standard deviation. The third worksheet shows the riboflavin calibration, with the R square calculation.

Supplementary data are named as follows in the Mendeley database [2]:

■ Supplementary Data 1_Luminescence.
■ Supplementary Data 2_Luminescence test tubes.
■ Supplementary Data 3_GFP.
■ Supplementary Data 4_Riboflavin.

## 2 Experimental Design, Materials, and Methods

### 2.1 Experimental Design

To construct the autoinduction device in *B. subtilis*, genes *luxR* and *luxI* from *Aliivibrio fischeri* and the respective promoters P*luxR* and P*luxI* were cloned into the pBS3Clux plasmid upstream of the luminescence operon *luxABCDE*. Genes *luxR* and *luxI* were named as induction module. The plasmid was then integrated into *B. subtilis* genome locus *sacA* as single copy. Reponse promoters R1 to R7 were introduced upstream of the *lux* operon. R1 and R6 promoters have also been used to control GFP expression in two configurations: *in cis*, when both the induction module and the response module (response promoter + *gfp*) were cloned into the same plasmid pBS1C and integrated at the *amy* locus in the genome; in trans, when the induction module was inserted at the *amy* locus using the plasmid pBS1C, and the response module inserted at the *lacA* locus in the chromosome using the plasmid pBS2E. GPF expression experiments have been performed using two different strains *B. subtilis* 168 and *B. subtilis* K07. Finally, the response promoter R6 was used to control the expression of the riboflavin operon *ribDGEABHT*. For that, the induction module and the rib operon under control of R6 were cloned into the pBS1C plasmid and inserted into the *B. subtilis* genome at the *amy* locus. All cloning steps have been performed using the Biobrick standard cloning.

**Figure 1:**
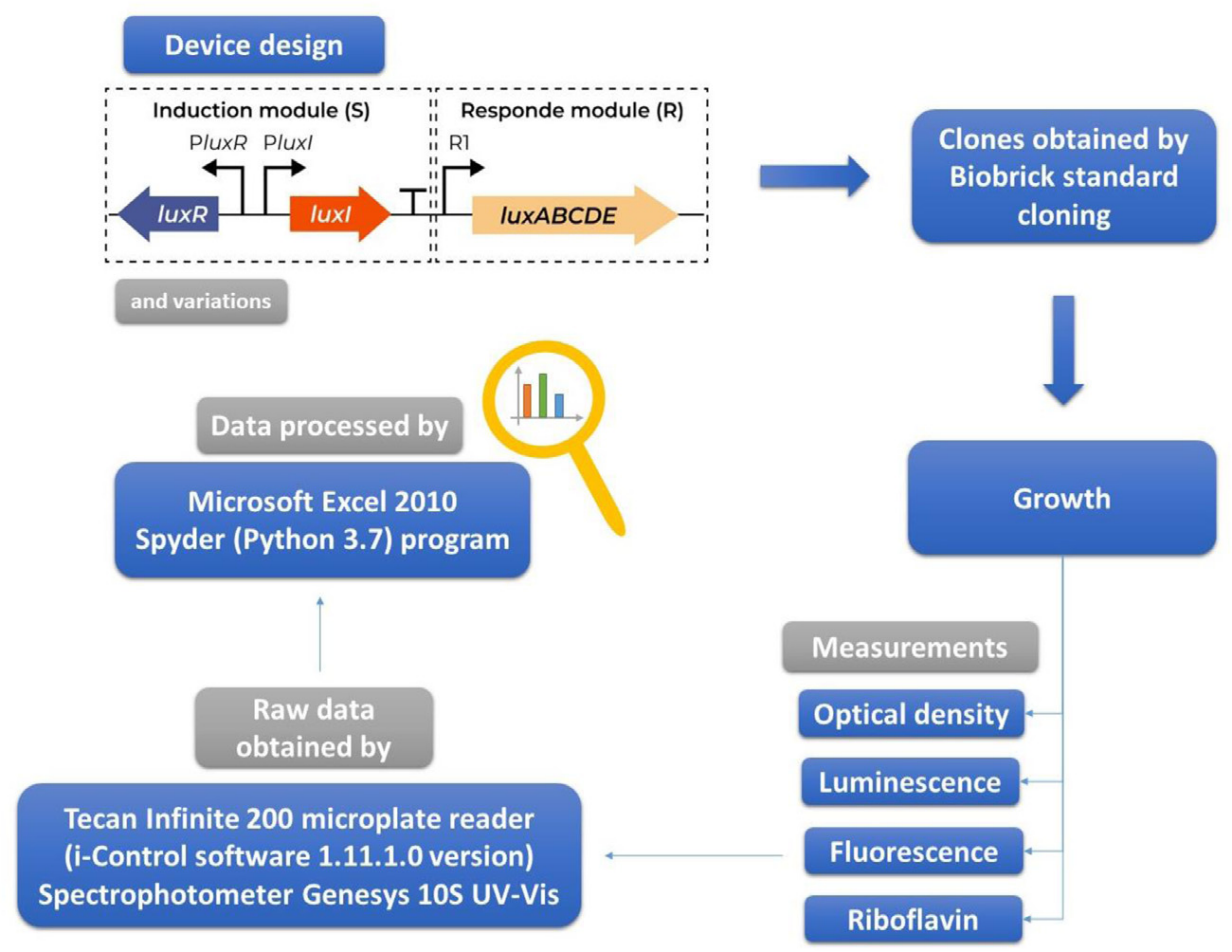
Summary of the experimental design.

The engineered strains were cultivated in 96-well microplates, test tubes or Erlenmeyer flasks, and periodic measurements were performed for optical density at 600 nm (OD_600_), luminescence and fluorescence. Figure 1 summarizes the experimental design.

### 2.2 Plasmids and other DNA sequences

All plasmids used were derived from the *Bacillus* BioBrick Box [3]: pBS3Clux (BBa_K823025), pBS2E (BBa_K823027), and pBS1C (BBa_K823023). Cloning work has been performed using the standard BioBrick cloning method. *luxR* and *luxI* genes and respective promoters were PCR amplified from genomic DNA from *A. fischeri. gfp* was PCR amplified from pDR111_GFP(Sp) (BGSCID ECE278) [4]. Riboflavin operon *ribDGEABHT* was PCR amplified from genomic DNA from *B. subtilis* starting 250 bp upstream of the *ribDG* start codon, ending 40 bp downstream of the *ribT* stop codon including the operon transcription terminator. The 250 bp upstream of *ribDG* includes a mutant version of the FMN riboswitch (GGGG to AAAA at position -217) known to prevent premature transcription termination driven by the riboswitch activity [5]. Synthetic sequences corresponding to promoters in the response modules were purchased as complementary oligonucleotides. A list of plasmids and strains used is provided in Table S1 in our main paper [1].

### 2.3 Bacterial strains and growth conditions

Bacterial strains used: *E. coli* Top10 for cloning and propagation of plasmids; *B. subtilis* 168, a wild type strain; and *B. subtilis* K07 (BGSCID 1A1133), a protease-free host.

Cultivation broths for *E. coli* strains: Lysogeny Broth (LB; 5 g L^−1^ yeast extract, 10 g L^−1^ tryptone, 10 g L^−1^ NaCl pH 7.2) supplemented with 100 µg mL^−1^ampicillin, when needed.

Cultivation broths for *B. subtilis* strains: LB broth supplemented with 5 µg mL^−1^chloramphenicol and/or 1 µg mL^−1^erythromycin, when needed. A high sugar complex medium was used for GFP measurements (PW broth: 70 g L^−1^ sucrose, 1 g L^−1^ yeast extract, 25 g L^−1^ NaNO_3_, 0.333 g L^−1^ KH_2_PO_4_, 1 g L^−1^ Na_2_HPO_4_•12H_2_O, 0.15 g L^−1^ MgSO4•7H_2_O, 7.5 mg L^−1^ CaCl_2_, 6 mgL^−1^ MnSO4•H_2_O, 6 mg L^−1^, FeSO_4_•7H_2_O, pH 7.0), and for some GFP measurements a mineral medium was used (MM: 4 g L^−1^ KH_2_PO_4_, 16 g L^−1^ K_2_HPO_4_•3H_2_O, 3.3 g L^−1^ (NH_4_)_2_SO_4_, 0.232 g L^−1^ MnSO_4_•4H_2_O, 12.3 g L^−1^ MgSO_4_•7H_2_O, 0,136 g L^−1^ ZnCl_2_, 0.05 g L^−1^ tryptophan, 0.022 g L^−1^ Iron ferric ammonium citrate, 5 g L^−1^ glucose).

Medium additives: N-(3-Oxohexanoyl)-L-homoserine lactone (Merk K3007) was added to the medium in concentrations from 0.01 to 100 nM, from an acetonitrile stock solution, for AHL-dependent induction of gene expression.

Microcultivation conditions: microcultivations were carried out on a microplate reader. The 96-well microplates were filled with 200 µL of culture medium, and incubated at 37°C and under 143 rpm orbital shaking.

Scale up conditions: test tubes 16 × 220 mm (d x L) were filled with 12 mL medium, and 500 mL baffled Erlenmeyer flasks filled with 50 mL LB broth were incubated at 37°C and 220 rpm.

All experiments were performed as three biological replicates.

### 2.4 Growth, luminescence and fluorescence measurements

Cell growth: culture optical density at 600 nm wavelength (OD_600_) was monitored on a 96-well microplate with optically clear bottom using a microplate reader, and on a 10 mm pathlength cuvette using a spectrophotometer.

Luminescence: measurements were carried out on a microplate reader using white microplates, and an integration time of 1,000ms.

GFP fluorescence: measurements were carried out on a microplate reader using black microplates, excitation at 485 nm, emission at 535 nm, and gain 100.

Riboflavin: riboflavin was quantified through luminescence on a microplate reader using black microplates. Measurements were carried out using excitation at 445 nm, and emission at 524 nm, and gain 80. A calibration curve was constructed using commercial riboflavin (Merck R7649). For riboflavin measurements in the culture medium, samples were periodically withdrawn from Erlenmeyer flasks, centrifuged at 12,000 xg for 5 min, and the flavin present in the supernatant were determined through fluorescence.

Normalization: luminescence, GFP fluorescence, and riboflavin concentration outputs were normalized to cell density by dividing each data-point by its corresponding OD_600_ value.

Maximal production: defined as the highest normalized output generated during cultivation (luminescence/OD_600_, fluorescence/OD_600_, and riboflavin/OD_600_).

Minimal production: defined as the lowest normalized output generated between time zero and the maximal production (minimal/initial). For identification of the minimal production, when the initial output was zero, the next sequence of outputs composed by at least four consecutive positive values was taken.

Switching OD_600_: the culture optical density (OD_600_), corresponding to the highest normalized production, such as luminescence/OD_600_, fluorescence/OD_600_, and riboflavin/OD_600_.

Fold change: defined as the ratio between the maximal and the minimal (initial) production of luminescence, fluorescence or riboflavin during cultivation. P_veg_ promoter was taken as reference for promoter strength receiving the arbitrary value 1.0. The ratio between the normalized production for all other promoters to P_veg_ was then calculated as relative promoter strength.

All raw and processed data files are available in Supplementary Data 1-4 [2].

## 3 Python processing and plotting

Graphics shown in the Supplementary material of the related research article [1] were generated using Spyder (Python 3.7). Microsoft excel datasheet containing raw data were imported to Python for processing and plotting. Luminescence and OD_600_ data were processed and plotted using the code bellow.

# Importing the libraries#

import numpy as np

import matplotlib.pyplot as plt

import pandas as pd

# Importing the dataset (Microsoft Excel Comma Separated Values File)

dsOD = pd.read_csv('OD .csv')

dsLum = pd.read_csv('Lum .csv')

# number of replicates

rep = 3

#OD_600_#

# Discounting the OD blank (LB)

OD = dsOD.iloc[:, 1:].values # excluding matrix time

brancoOD = np.mean(OD[:, 0:3])

OD = np.delete(OD, [0,1,2], 1) # deleting columns with LB data

ODbg = OD-brancoOD # discounting the OD values blank

# Calculating mean and deviation of OD

col = np.size(ODbg, 1) # number of columns

lin = np.size(ODbg, 0) # number of lines

#lin = 41

colMedia = int(col/rep)

dataRep = np.zeros((1,rep)) # stores data to calculate averages

# OD calculations

mediaOD = np.zeros((lin, colMedia))

desvOD = np.zeros((lin, colMedia))

for i in range(0, lin):

    ct = 0

    for j in range(0, col, rep):

        limite = j+(rep)

        dataCounter = 0

        for k in range(j, limite):

            dataRep[0, dataCounter] = ODbg[i,k]

            dataCounter = dataCounter + 1

        mediaOD[i, ct] = np.mean(dataRep)

        desvOD[i,ct] = np.std(dataRep)

        dataRep[:,:] = 0

        ct = ct+1

#Luminescence#

i = 0

j = 0

k = 0

ct = 0

limite = 0

dataCounter = 0

# Discounting the luminescence blank (Negative Control)

# Excluding time and LB from the Lum matrix

Lum = dsLum.iloc[:, 4:].values

# Indicate the values of the negative control

# column value where negative control values start: column value where it ends + 1

BrancoLum = Lum[:, 3:6]

MediaBrancoLum = np.zeros((lin, 1))

# Blank point-to-point average

for i in range(0, lin):

    MediaBrancoLum[i, 0] = np.mean(BrancoLum[i, :])

# Luminescence calculations and discounting blank

Lumbg = np.zeros((lin, col))

mediaLum = np.zeros((lin, colMedia))

desvLum = np.zeros((lin, colMedia))

# Discount the luminescence blank

for i in range(0, lin):

    for j in range(0, col):

        Lumbg[i,j] = Lum[i,j] - MediaBrancoLum[i, 0]

# Replace negatives with 0 in luminescence

for i in range(0, lin):

    for j in range(0, col):

        if Lumbg[i,j] < 0:

            Lumbg[i,j] = 0

# Calculating mean and deviation

for i in range(0, lin):

    ct = 0

    for j in range(0, col, rep):

        limite = j+(rep)

        dataCounter = 0

        for k in range(j, limite):

            dataRep[0, dataCounter] = Lumbg[i,k]

            dataCounter = dataCounter + 1

        mediaLum[i, ct] = np.mean(dataRep)

        desvLum[i, ct] = np.std(dataRep)

        dataRep[:,:] = 0

        ct = ct+1   

mediaLum[np.isnan(mediaLum)] = 0

desvLum[np.isnan(desvLum)] = 0

#PLOT #

# Selecting microorganism

for MO in range(0, colMedia):

    #MO = 2

    strainOD = mediaOD[:, MO]

    desvioOD = desvOD[:, MO]

    strainLum = mediaLum[:, MO]

    desvioLum = desvLum[:, MO]

    # Let's plot!

    fig, ax1 = plt.subplots()

    ax1.errorbar(strainOD, strainLum, yerr=desvioLum, xerr=desvioOD, fmt='o', color='k', markersize=4, capsize=3, elinewidth=0.5)

    ax1.set_xlabel('OD600′, fontsize='18′, labelpad=10, fontweight='bold', fontfamily='arial')

    ax1.set_ylabel('Bioluminescence (a.u.)', fontsize='18′, labelpad=10, fontweight='bold', fontfamily='arial')

    ax1.tick_params(axis='y', labelsize='16′)

    ax1.tick_params(axis='x', labelsize='16′)

    ax1.set_xticks([0, 0.1, 0.2, 0.3, 0.4, 0.5, 0.6, 0.7, 0.8])

    ax1.set_xlim(0, 0.8)

    ax1.set_ylim(0,)

    fig.tight_layout()

    plt.show()

## Declaration of Competing Interest

The authors declare that they have no known competing financial interests or personal relationships which have, or could be perceived to have, influenced the work reported in this article.

## Appendix Supplementary materials

Image, application 1

## References

[1] Correa G.G., Lins M.R.C.R., Silva B.F., Paiva G.B., Zocca V.F.B., Ribeiro N.V., Picheli F.P., Mack M., Pedrolli D.B. (2020). A modular autoinduction device for control of gene expression in *Bacillus subtilis*. Metab. Eng..

[2] Correa G.G., Lins M.R.C.R., Silva B.F., Paiva G.B., Zocca V.F.B., Ribeiro N.V., Picheli F.P., Mack M., Pedrolli D.B. (2020). Dataset for supporting a modular autoinduction device for control of gene expression in *Bacillus subtilis, Mendeley Data*. v2.

[3] Radeck J., Kraft K., Bartels J., Cikovic T., Durr F., Emenegger J., Kelterborn S., Sauer C., Fritz G., Gebhard S., Mascher T. (2013). The Bacillus BioBrick Box: generation and evaluation of essential genetic building blocks for standardized work with *Bacillus subtilis*. J. Biol. Eng..

[4] Overkamp W., Beilharz K., Detert Oude Weme R., Solopova A., Karsens H., Kovacs A., Kok J., Kuipers O.P., Veening J.W. (2013). Benchmarking various green fluorescent protein variants in *Bacillus subtilis, Streptococcus pneumon*iae, and *Lactococcus lactis* for live cell imaging. Appl. Environ. Microbiol..

[5] Winkler W.C., Cohen-Chalamish S., Breaker R.R. (2002). An mRNA structure that controls gene expression by binding FMN. Proc. Natl. Acad. Sci. U S A..

